# A meta-analysis of gemcitabine containing chemotherapy for locally advanced and metastatic pancreatic adenocarcinoma

**DOI:** 10.1186/1756-8722-4-11

**Published:** 2011-03-26

**Authors:** Jing Hu, Gang Zhao, Hong-Xia Wang, Lei Tang, Ying-Chun Xu, Yue Ma, Feng-Chun Zhang

**Affiliations:** 1 Department of Oncology, Shanghai Renji Hospital, Shanghai Jiaotong University School of Medicine, Shanghai 200127, China; 2 Department of Surgery, Shanghai Renji Hospital, Shanghai Jiaotong University School of Medicine, Shanghai 200127, China; 3 Department of Oncology, Suzhou Kowloon Hospital, Shanghai Jiaotong University School of Medicine, Suzhou 215021, China

**Keywords:** gemcitabine, chemotherapy, pancreatic adenocarcinoma

## Abstract

**Background:**

The objectives of the present study are to investigate the efficacy and safety profile of gemcitabine-based combinations in the treatment of locally advanced and metastatic pancreatic adenocarcinoma (LA/MPC).

**Methods:**

We performed a computerized search using combinations of the following keywords: "chemotherapy", "gemcitabine", "trial", and "pancreatic cancer".

**Results:**

Thirty-five trials were included in the present analysis, with a total of 9,979 patients accrued. The analysis showed that the gemcitabine-based combination therapy was associated with significantly better overall survival (OS) (ORs, 1.15; p = 0.011), progression-free survival (PFS) (ORs, 1.27; p < 0.001), and overall response rate (ORR) (ORs, 1.58; p < 0.001) than gemcitabine monotherapy. Similar results were obtained when the gemcitabine-fluoropyrimidine combination was compared with gemcitabine, with the OS (ORs, 1.33; p = 0.007), PFS (ORs, 1.53; p < 0.001), and ORR (ORs 1.47, p = 0.03) being better in the case of the former. The OS (ORs, 1.33; p = 0.019), PFS (ORs, 1.38; p = 0.011), and one-year survival (ORs, 1.40; p = 0.04) achieved with the gemcitabine-oxaliplatin combination were significantly greater than those achieved with gemcitabine alone. However, no survival benefit (OS: ORs, 1.01, p = 0.93; PFS: ORs, 1.19, p = 0.17) was noted when the gemcitabine-cisplatin combination was compared to gemcitabine monotherapy. The combinations of gemcitabine and other cytotoxic agents also afforded disappointing results. Our analysis indicated that the ORR improved when patients were treated with the gemcitabine-camptothecin combination rather than gemcitabine alone (ORs, 2.03; p = 0.003); however, there were no differences in the OS (ORs, 1.03; p = 0.82) and PFS (ORs, 0.97; p = 0.78) in this case.

**Conclusions:**

Gemcitabine in combination with capecitabine or oxaliplatin was associated with enhanced OS and ORR as compared with gemcitabine in monotherapy, which are likely to become the preferred standard first-line treatment of LA/MPC.

## Background

Pancreatic adenocarcinoma is the fifth leading cause of death due to solid tumors in Western industrialized countries. Because pancreatic adenocarcinoma is often difficult to detect in early stages, most patients are diagnosed with advanced or metastatic disease at first presentation [1,2]. The median survival of patients with locally advanced disease is 6 to 10 months, compared to 3 to 6 months for patients with metastatic disease [3].

Gemcitabine (Gemzar™; 2',2'-difluorodeoxycytidine) is a pyrimidine antimetabolite and a specific analogue of deoxycytidine. At present, gemcitabine monotherapy remains the standard care for patients with locally advanced and metastatic pancreatic adenocarcinoma (LA/MPC) [4]. However, patients who receive this therapy have a median overall survival (OS) of only 5.65 months [5]. In an effort to increase the objective response rate (RR) and survival of LA/MPC patients, many trials have been carried out in the last ten years to evaluate gemcitabine monotherapy or combination therapy regimens. Currently, the National Comprehensive Cancer Network (NCCN) guidelines indicate that gemcitabine combined with one other agent is the optimal treatment for LA/MPC patients with evidence of category 2B disease (recommendation based on lower-level evidence).

It is unclear whether this regimen is the ideal treatment for LA/MPC or whether it should be reevaluated. Therefore, we undertook a systematic review and quantitative meta-analysis to evaluate the available evidence from relevant randomized trials. This review will summarize the various trials of gemcitabine-based chemotherapy regimens in LA/MPC and discuss how these results should affect clinical practice.

## Methods

### Search strategy

We carried out a comprehensive search of the literature for randomized controlled trials in Pubmed using the terms "chemotherapy," "gemcitabine," "trials," and "pancreatic cancer" (no limitation for language). In addition to full publications, abstracts presented at the annual meetings of the American Society of Clinical Oncology (ASCO) and the European Cancer Conference (ECCO) were included.

### Selection criteria

To be eligible for inclusion, trials were required to be prospective, properly randomized and well designed, which we defined as matched for age, stage and performance status (PS) or Karnofsky performance status (KPS). Patients with locally advanced or metastatic disease were included in the study, and histologic or cytologic confirmation of pancreatic adenocarcinoma was required.

If a trial included concomitant interventions such as radiotherapy or radioisotope treatment that differed systematically between the investigated arms, the trial was excluded. Whenever we encountered reports pertaining to overlapping patient populations, we included only the report with longest follow-up (having the largest number of events) in the analysis. Only randomized trials were included, and randomization must have started on or after Jan 1, 1965. The deadline for eligible trial publication was July 30, 2010.

### Data collection

Two reviewers (Jing Hu and Gang Zhao) assessed the identified abstracts. Both reviewers independently selected trials for inclusion according to prior agreement regarding the study population and intervention. Lei Tang and Ying-Chun Xu also cross-checked all data collected against the original articles. If one of the reviewers determined that an abstract was eligible, the full text of article was retrieved and reviewed in detail by all reviewers.

For the 35 trials included in the meta-analysis, we gathered the authors' names, journal, year of publication, sample size (randomized and analyzed) per arm, performance status, regimens used, line of treatment, median age of patients and information pertaining to study design (whether the trial reported the mode of randomization, allocation concealment, description of withdrawals per arm and blinding).

### Statistical analysis

The meta-analysis was performed using Review Manager Version 4.2 (Nordic Cochran Centre, Copenhagen) and Comprehensive Meta Analysis Version 2 (Biostat™, Englewood, NJ). Heterogeneity between the trials was assessed to determine which model should be used. To assess statistical heterogeneity between studies, the Cochran Q test was performed with a predefined significance threshold of 0.05. Odds ratios (ORs) were the principal measurements of effect and were presented with a 95% confidence interval (CI). P values of < 0.05 were considered statistically significant. All reported p-values result from two-sided versions of the respective tests. The revision of funnel plots did not reveal any considerable publication bias.

The primary outcome measurements were overall survival (OS) and progression-free survival (PFS, time from randomization to progression or death), and secondary endpoints were overall response rate (ORR, number of partial and complete responses) and toxicity. Toxicities recorded by the original research group were recorded in our analysis, and the most frequent events were analyzed. In order to optimize our assessment of response, we used trials that included patients with measurable or assessable diseases and that were analyzed predominantly according to the World Health Organization (WHO) criteria. Toxicity profiles were reported according to the WHO criteria.

## Results

### Selection of the trials

**Figure 1:**
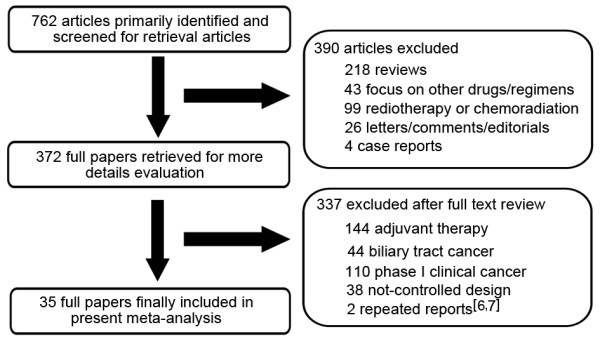
**Flow chart for trials selection in the meta-analysis**.

The literature search uncovered 762 articles. Primary screening led to the exclusion of 390 articles for the following reasons: reviews (218), other agents/regimens (43), radiotherapy/chemoradiation (99), letters/comments/editorials [26] or case reports [4]. The remaining 372 papers were retrieved for more detailed evaluation. Of these, 144 articles were excluded because of adjuvant chemotherapy, 44 for biliary tract cancer, 110 for phase I clinical trials, 38 for not-controlled design and 2 for repeated reports [6,7]. In the end, a total of 35 randomized clinical trials [8-42] were eligible for inclusion in our analysis (Figure 1).

### Characteristics of the trials included in the present analysis

Thirty-five trials were included in the present analysis, with a total of 9, 979 patients accrued. Characteristics of the eligible trials are listed in Table 1. Most of the trials (34/35, 97%) evaluated gemcitabine-based chemotherapy for first line or palliative chemotherapy in LA/MPC patients, whereas one trial (Palmer 2007) evaluated neoadjuvant chemotherapy. Twenty-three trials compared single-agent gemcitabine with gemcitabine combined with other cytotoxic agents, nine trials studied gemcitabine monotherapy with gemcitabine plus targeted therapy, and three trials evaluated triplet therapy for LA/MPC patients.

**Table 1:** Characteristics of the eligible trials included in the meta-analysis

Trial	No. of pts		Regimens (per arm)	No. of pts (per arm)	Male	Median age (range)(y)	PS 0-2/KPS≥50	M*
Gong JF	40	palliative	Gem-X**	25	56%	63 (45-76)	UK	UK
2007[8]			Gem	15	66.7%	63 (45-76)		
Reni M	104	first line	PEFG	52	46.2%	62 (37-69)	100%	71%
2005[9]			Gem	47	40.7%	59 (25-69)	100%	56%
Gem versus Gem plus fluoropyrimidine								
Cunningham D	533	first line	Gem/Cap	267	60%	62 (37-82)	100%	70%
2009[10]			Gem	266	58%	62 (26-83)	100%	71%
Bernhard J	319	palliative	Gem/Cap	160	54%	62 (27-83)	100%	80%
2008[11]			Gem	159	53%	62 (36-84)	100%	79%
Scheithauer W	83	first line	Gem/Cap	41	66%	64 (40-75)	100%	UK
2003[12]			Gem	42	55%	66 (39-75)	100%	
Berlin JD	327	first line	Gem/5-FU	160	51.8%	65.8 (28-84)	100%	89.4%
2002[13]			Gem	162	53.7%	64.3 (33-85)	100%	90.1%
Di Costanzo F	94	first line	Gem/5-FU	45	63%	62 (44-75)	100%	67%
2005[14]			Gem	49	48%	64 (34-75)	100%	73%
Riess H[[Bibr B15]]	473	first line	Gem/5-FU	235	UK	UK	100%	UK
2005			Gem	238			100%	
Gem versus Gem plus platinum								
Louvet C	313	first line	Gem/Oxa	157	60%	61 (35-77)	100%	68%
2005[16]			Gem	156	53%	60 (22-75)	100%	70%
Poplin E	824	first line	Gem/Oxa	272	45.6%	63 (29-96)	99.6%	89.3%
2009[17]			Gem	275	56.4%	63 (31-88)	100%	90.2%
			Gem FDR	277	57.8%	62 (36-87)	99.6%	88.8%
Yan ZC	60	first line	Gem/Oxa	30	63.3%	58 (23-75)	31.7%	UK
2007[18]			Gem	30	63.3%	58 (23-75)	31.7%	UK
Colucci G	400	first line	Gem/DDP	201	62.2%	63 (35-75)	100%	84.6%
2010[19]			Gem	199	56.8%	63 (37-75)	100%	82.9%
Colucci G	107	first line	Gem/DDP	53	66%	60 (33-71)	100%	62%
2002[20]			Gem	54	50%	63 (43-75)	100%	54%
Wang XY	42	first line	Gem/DDP	22	68.2%	65 (37-76)	100%	68.2%
2002[21]			Gem	20	70.0%	57 (35-60)	100%	50%
Heinemann V	195	first line	Gem/DDP	98	65.3%	64 (37-82)	100%	80%
2006[22]			Gem	97	61.9%	66 (43-85)	100%	78.9%
Palmer DH	50	neoadjuvant	Gem/DDP	26	50%	66 (47-78)	100%	UK
2007[23]			Gem	24	54%	66 (40-79)	100%	
Li CP	46	first line	Gem/DDP	21	UK	UK	UK	UK
2004[24]			Gem	25				
Kulke MH	259	first line	Gem/DDP	66	56%	59 (36-84)	100%	UK
2009[25]			Gem FDR	64	66%	59 (31-81)	100%	UK
			Gem/Doc	65	62%	63 (41-79)	100%	UK
			Gem/CPT-11	64	68%	61 (32-77)	100%	UK
Viret F	83	first line	Gem/DDP	42	UK	62	100%	81%
2004[26]			Gem	41	UK	63	100%	78%
Gem versus camptothecin								
Stathopoulos GP	130	first line	Gem/CPT-11	60	65%	64 (31-84)	100%	78%
2006[27]			Gem	70	60%	64 (44-83)	100%	86%
Rocha Lima CM	360	first line	Gem/CPT-11	180	57.2%	63 (39-81)	97.2%	82.2%
2004[28]			Gem	180	53.3%	60 (32-83)	93.9%	80.6%
Abou-Alfa GK	349	first line	Gem/exatecan	175	53%	63 (36-85)	99%	79%
2006[29]			Gem	174	57%	62 (30-84)	100%	78%
Gem versus pemetrexed								
Oettle H	565	palliative	Gem/Pem*	283	60.4%	63 (27-82)	98.9%	90.1%
2005[30]			Gem	282	53.5%	63 (28-82)	98.9%	91.1%
Gem versus Gem plus targeted therapy								
Moore MJ	569	palliative	Gem/erlotinib	285	47.7%	64 (38-84)	99.6%	76.5%
2007[31]			Gem	284	57%	64 (36-92)	100%	75%
Van Cutsem E	688	first line	Gem/tipifarnib	341	57%	61 (29-89)	100%	76%
2004[32]			Gem	347	58%	62 (30-88)	100%	77%
Philip PA	743	palliative	Gem/Cetuximab	372	51%	63.7	100%	79%
2010[33]			Gem	371	54%	64.3	100%	78%
Saif MW	135	palliative	Gem/LY293111	67	60%	62(33-82)	99%	87%
2009[34]			Gem	66	60%	62(34-85)	99%	90%
Spano JP	103	palliative	Gem/axitinib	69	51%	65(44-81)	100%	58%
2008[35]			Gem	34	47%	61(36-78)	100%	56%
Bramhall SR	239	first line	Gem/marimastat	120	57.5%	62 (32-83)	100%	59%
2002[36]			Gem	119	59.7%	62 (37-85)	100%	62%
Kindler HL	602	first line	Gem/Bev	302	58%	64 (26-88)	100%	84%
2010[37]			Gem	300	51%	65 (35-86)	100%	85%
Richards DA	174	first line	Gem/CI-994	86	59.3%	62 (32-82)	100%	82.6%
2006[38]			Gem	88	60.2%	65 (36-83)	100%	83%
Friess H	89	first line	Gem/Cilengitide	46	57%	68 (40-80)	100%	93%
2006[39]			Gem	43	42%	66 (56-80)	100%	90%
the others								
Cascino S	84	first line	C-225/Gem/DDP	42	69%	61 (38-78)	100%	73.8%
2008[40]			Gem/DDP	42	52%	64 (40-76)	100%	71.4%
Vervenne W	607	first line	Gem/erlotinib/Bev	306	57%	62	100%	100%
2008[41]			Gem/erlotinib	301	62%	61	100%	100%
Boeck S	190	first line	Cap/Oxa	61	65%	62 (37-74)	100%	63%
2007[42]			Gem/Cap	64	57%	63 (47-75)	100%	69%
			Gem/Oxa	63	70%	63 (45-75)	100%	71%

Note: Gem, gemcitabine; DDP, cisplatin; C-225, Cetuximab; Bev, bevacizumab; Oxa, oxaliplatin; Cap, capecitabine; Doc, docetaxel; FDR, fixed dose rate; UK, unknown; M*, metastatic disease; Gem-X, gemcitabine combined with 5-FU or capecitabine or cisplatin or oxaliplatin.

Among the thirty-five trials, the distribution of baseline patient characteristics was homogeneous. The percentage of patients with metastatic disease ranged from 50% to 91.1%, while the median age of patients varied from 57.8 to 66 (range: 23-96). The details of chemotherapeutic regimens per arm in each trial are shown in Table 2.

**Table 2:** Regimens of the trials included in this analysis.

Trial	Arm	Regimens
Gong JF 2007	Gem/X	Gem 1,000 mg/m^2 ^d_1,8_; 5-FU 425-600 mg/m^2 ^d_1-5_, or DDP 30-37.5 mg/m^2 ^d_1-2_, or Oxa 85-130 mg/m^2 ^d_1_, or Cap 1 000 mg/m^2 ^bid d_1-14_, q3w.
	Gem	Gem 1,000 mg/m^2 ^weekly × 7 followed by a 2-week rest, then weekly for 3 weeks, q4w.
Reni M 2005	PEFG	DDP 40 mg/m^2 ^d_1_, EPI 40 mg/m^2 ^d_1_, Gem 600 mg/m^2 ^d_1,8_, 5-FU 200 mg/m^2 ^d_1-28_, q4w.
	Gem	Gem 1,000 mg/m^2 ^weekly × 7 followed by a 2-week rest, then weekly for 3 weeks, q4w.
Gem versus Gem plus fluoropyrimidine		
Cunningham D	Gem/Cap	Gem 1,000 mg/m^2 ^weekly for 3 weeks; Cap 830 mg/m^2 ^bid po for 3 weeks, q4w
2009	Gem	Gem 1,000 mg/m^2 ^weekly × 7 followed by 1-week rest, then weekly for 3 weeks, q4w.
Bernhard J 2008	Gem/Cap	Gem 1,000 mg/m^2 ^d_1,8_; Cap 650 mg/m^2 ^bid po d_1-14_, q3w.
	Gem	Gem 1,000 mg/m^2 ^weekly × 7 followed by 1-week rest, then weekly for 3 weeks, q4w.
Scheithauer	Gem/Cap	Gem 2200 mg/m^2 ^d_1_, Cap 2500 mg/m^2 ^d_1-7_, q2w.
W 2003	Gem	Gem 2200 mg/m^2 ^d_1_, q2w.
Berlin JD 2002	Gem/5-FU	Gem 1,000 mg/m^2 ^weekly, 5-FU 600 mg/m^2 ^weekly for 3 weeks, q4w.
	Gem	Gem 1,000 mg/m^2 ^weekly for 3 weeks, q4w.
Di Costanzo	Gem/5-FU	Gem was combined with 5-FU 200 mg/m^2 ^for 6 weeks in the first cycle, followed by a week of rest; then for 3 weeks, q4w.
F 2005	Gem	Gem 1,000 mg/m^2 ^weekly × 7 followed by a 2-week rest, then weekly for 3 weeks, q4w.
Riess H 2005	GFF	Gem 1,000 mg/m^2^, 5-FU 750 mg/m^2^, folinic acid 200 mg/m^2 ^d_1,8,15,22_, q6w.
	Gem	Gem 1,000 mg/m^2 ^weekly × 7 followed by a 2-week rest, then weekly for 3 weeks, q4w.
Gem versus Gem plus platinum		
Louvet C 2005	Gem/Oxa	Gem 1,000 mg/m^2 ^d_1_, Oxa 100 mg/m^2 ^d_2_, q2w.
	Gem	Gem 1,000 mg/m^2 ^weekly × 7 followed by 1-week rest, then weekly for 3 weeks, q4w.
Poplin E 2009	Gem/Oxa	Gem 1,000 mg/m^2 ^d_1_, Oxa100 mg/m^2 ^d_2_, q2w.
	Gem	Gem 1,000 mg/m^2 ^weekly × 7 followed by 1-week rest, then weekly for 3 weeks, q4w.
	Gem FDR	Gem 1,500 mg/m^2 ^administered as a 150 minutes infusion d_1,8,15_, q4w.
Yan ZC 2007	Gem/Oxa	Gem 1,000 mg/m^2 ^d_1_, Oxa 100 mg/m^2 ^d_2_, q2w.
	Gem	Gem 1,000 mg/m^2 ^d_1,8,15_, q4w.
Colucci G 2010	Gem/DDP	Gem 1,000 mg/m^2 ^weekly × 7 followed by 1-week rest, then weekly for 3 weeks, q4w; DDP 25 mg/m^2 ^added weekly to Gem.
	Gem	Gem 1,000 mg/m^2 ^weekly × 7 followed by 1-week rest, then weekly for 3 weeks, q4w.
Colucci G 2002	Gem/DDP	Gem 1000 mg/m^2 ^weekly × 7 followed by 2-week rest, DDP 25 mg/m^2 ^per week 1 hour before Gem.
	Gem	Gem 1,000 mg/m^2 ^weekly × 7 followed by 2-week rest, then weekly for 3 weeks, q4w.
Wang XY 2002	Gem/DDP	Gem 1 000 mg/m^2 ^d_1,8,15_; DDP 60 mg/m^2 ^on d_15_, q4w.
	Gem	Gem 1,000 mg/m^2 ^weekly × 7 followed by 1-week rest, then weekly for 3 weeks, q4w.
Heinemann V 2006	Gem/DDP	Gem 1,000 mg/m^2^, DDP 50 mg/m^2 ^d_1,15_, q4w.
	Gem	Gem 1,000 mg/m^2 ^d_1,8,15_, q4w.
Palmer DH 2007	Gem/DDP	Gem 1000 mg/m^2 ^every 7 days for 43 days, followed immediately by DDP 25 mg/m^2^
	Gem	Gem 1000 mg/m^2 ^every 7 days for 43 days
Li CP 2004	Gem/DDP	Gem 1000 mg/m^2^/week and DDP 25 mg/m^2^/week × 3 every 4 weeks
	Gem	Gem 1000 mg/m^2 ^× 3 every 4 weeks
Kulke MH 2009	Gem/DDP	Gem 1,000 mg/m^2 ^d_1,8,15_; DDP 50 mg/m^2 ^d_1,15_, q4w.
	Gem FDR	Gem 1,500 mg/m^2 ^at a rate of 10 mg/m^2^/min d_1,8,15_, q4w.
	Gem/Doc	Gem 1,000 mg/m^2^; Doc 40 mg/m^2 ^d_1,8_, q3w.
	Gem/CPT-11	Gem 1,000 mg/m^2^; irinotecan 100 mg/m^2 ^d_1,8_, q3w.
Viret F 2004	Gem/DDP	Gem 1000 mg/m^2 ^d_1,8,15_; DDP 75 mg/m^2 ^d_15_, q4w.
	Gem	Gem 1000 mg/m^2 ^weekly × 7 followed by 1 week of rest, then weekly for 3 weeks, q4w
Gem versus camptothecin		
Stathopoulos GP 2006	Gem/CPT-11	Gem d_1,8_; CPT-11 300 mg/m^2 ^d_8_, q3w.
	Gem	Gem 900 mg/m^2 ^d_1,8,15_, q4w.
Rocha Lima CM 2004	Gem/CPT-11	Gem 1,000 mg/m^2 ^and CPT-11 100 mg/m^2 ^given weekly for 2 weeks every 3-week cycle.
	Gem	Gem 1,000 mg/m^2 ^weekly × 7 followed by 1-week rest, then weekly for 3 weeks, q4w.
Abou-Alfa GK 2006	Gem/Exat	Exatecan 2.0 mg/m^2 ^and Gem 1,000 mg/m^2 ^were administered on days 1 and 8, q3w.
	Gem	Gem 1,000 mg/m^2 ^weekly × 7 followed by 1-week rest, then weekly for 3 weeks, q4w.
Gem versus pemetrexed		
Oettle H 2005	Gem/Pem	Gem 1,250 mg/m^2 ^d_1,8_; pemetrexed 500 mg/m^2 ^d_8_, q3w.
	Gem	Gem 1,000 mg/m^2 ^d_1,8,15_, q4w.
Gem versus Gem plus targeted therapy		
Moore MJ 2007	Gem/Erlo	Gem 1,000 mg/m^2 ^weekly × 7 followed by 1-week rest, then weekly for 3 weeks, q4w; Erlotinib 100 or 150 mg/d po
	Gem	Gem 1,000 mg/m^2 ^weekly × 7 followed by 1-week rest, then weekly for 3 weeks, q4w
Van Cutsem E 2004	Gem/Tipi	Gem 1,000 mg/m^2 ^weekly × 7 followed by 1-week rest, then weekly for 3 weeks, q4w; Tipifarnib 200 mg bid po continuously;
	Gem	Gem 1,000 mg/m^2 ^weekly × 7 followed by 1-week rest, then weekly for 3 weeks, q4w
Philip PA 2010	Gem/C-225	Gem 1,000 mg/m^2 ^weekly × 7 followed by 1-week rest, then weekly for 3 weeks, q4w;
		Cetuximab 400 mg/m^2 ^on week 1, followed by weekly 250 mg/m^2^.
	Gem	Gem 1,000 mg/m^2 ^weekly × 7 followed by 1-week rest, then weekly for 3 weeks, q4w
Saif MW 2009	Gem/LY	Gem 1000 mg/m^2 ^d_1,8,15_, q4w; continuously administered LY 600 mg twice daily.
	Gem	Gem 1000 mg/m^2 ^d_1,8,15_, q4w.
Spano JP 2008	Gem/Axitinib	Gem 1000 mg/m^2 ^d_1,8,15_, q4w; Axitinib 5 mg twice daily.
	Gem	Gem 1000 mg/m^2 ^d_1,8,15_, q4w.
Bramhall SR 2002	Gem/Marimastat	Gem 1,000 mg/m^2 ^weekly × 7 followed by 1-week rest, then weekly for 3 weeks, q4w; Marimastat 25 mg bid po.
	Gem	Gem 1,000 mg/m^2 ^weekly × 7 followed by 1-week rest, then weekly for 3 weeks, q4w.
Kindler HL 2010	Gem/Bev	Gem 1,000 mg/m^2 ^d_1,8,15_; Bev 10 mg/kg d_1,15_; q4w.
	Gem	Gem 1,000 mg/m^2 ^d_1,8,15_; q4w.
Richards DA 2006	Gem/CI-994	Gem 1000 mg/m^2 ^d_1,8,15_; CI-994 6 mg/m^2 ^d_1-21_; q4w.
	Gem	Gem 1000 mg/m^2 ^d_1,8,5_; q4w.
Friess H 2006	Gem/Cile	Gem 1000 mg/m^2 ^d_1,8,15_; Cilengitide 600 mg/m^2 ^twice weekly; q3w.
	Gem	Gem 1000 mg/m^2 ^d_1,8,15_; q3w.
the others		
Cascino S 2008	C-225/Gem/DDP	Cetuximab 250 mg/m^2 ^weekly, after a loading dose of 400 mg/m2; Gem 1000 mg/m^2 ^and DDP 35 mg/m^2 ^on d_1,8_; q3w.
	Gem/DDP	Gem 1000 mg/m^2 ^and DDP 35 mg/m^2 ^on d_1,8_; q3w.
Vervenne W 2008	Gem/Erlo/Bev	Gem 1,000 mg/m^2 ^weekly × 7 during first 8 weeks, then for 3 weeks, q4w.
		Erlotinib 100 mg/d po daily; Bevacizumab 5 mg/kg q2w.
	Gem/Erlo	Gem 1,000 mg/m^2 ^weekly × 7 for 7 weeks followed by 1-week rest, then weekly for 3 weeks, q4w; Erlotinib 100 mg/d po daily.
Boeck S 2007	Cap/Oxa	Cap 1000 mg/m^2 ^bid d_1-14_; Oxa 130 mg/m^2 ^d_1._
	Gem/Cap	Gem 1,000 mg/m^2 ^d_1,8_; Cap 825 mg/m^2 ^bid d_1-14_
	Gem/Oxa	Gem 1,000 mg/m^2 ^d_1,8_; Oxa 130 mg/m^2 ^d_8_

Note: Gem, gemcitabine; DDP, cisplatin; 5-FU, 5-fluorouracil; EPI, epirubicin; CPT-11, irinotecan; Bev, bevacizumab; Oxa, oxaliplatin; Cap, capecitabine; Doc, docetaxel; FDR, fixed dose rate; Exat, exatecan; Tipi, tipifarnib; Erlo, Erlotinib; C-225, cetuximab; Cile, Cilengitide; Bev, bevacizumab; LY, LY293111; UK, unknown; M*, metastatic disease; Gem-X, gemcitabine combined with 5-FU or capecitabine or cisplatin or oxaliplatin.

### Trials comparing single-agent gemcitabine with gemcitabine combined with other cytotoxic agents

**Figure 2:**
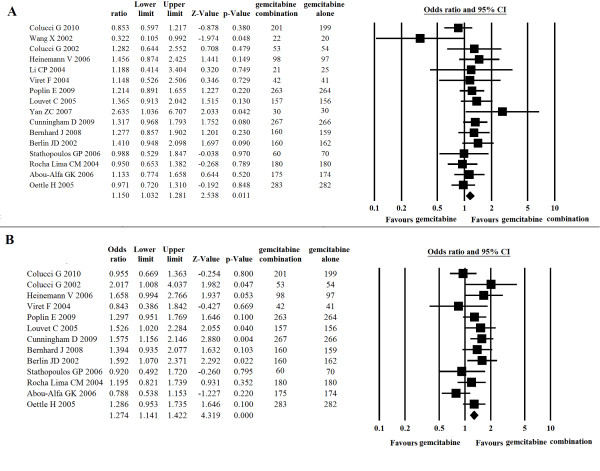
**Comparison of gemcitabine-X combination with gemcitabine alone**. A, OS; B, PFS.

This analysis evaluated 23 trials (5,577 patients) comparing single-agent gemcitabine with gemcitabine-based combinations with other cytotoxic agents. For the primary endpoint of OS, the gemcitabine-based combination therapy was associated with significantly better outcome (ORs, 1.15; 95% CI, 1.03-1.28; p = 0.011) than gemcitabine in monotherapy (Figure 2A). The analysis of PFS also afforded favorable results for the combination arm, with the ORs being 1.27 (95% CI, 1.14-1.42; p < 0.001) (Figure 2B). A similar advantage for gemcitabine-based combinations was observed in terms of the ORR (ORs, 1.58; 95% CI, 1.31-1.91; p < 0.001), with no significant heterogeneity (p = 0.79).

### Trials comparing gemcitabine alone with gemcitabine plus fluoropyrimidine

Six studies involving 1829 patients (Cunningham 2009, Bernhard 2008, Scheithauer 2003, Berlin 2004, Di Costanzo 2005, Riess 2005) compared single agent gemcitabine with gemcitabine plus fluoropyrimidine. Both oral capecitabine and infused 5-fluorouracil (5-FU) were evaluated in combination with gemcitabine in a variety of dosing schedules in these studies.

**Figure 3:**
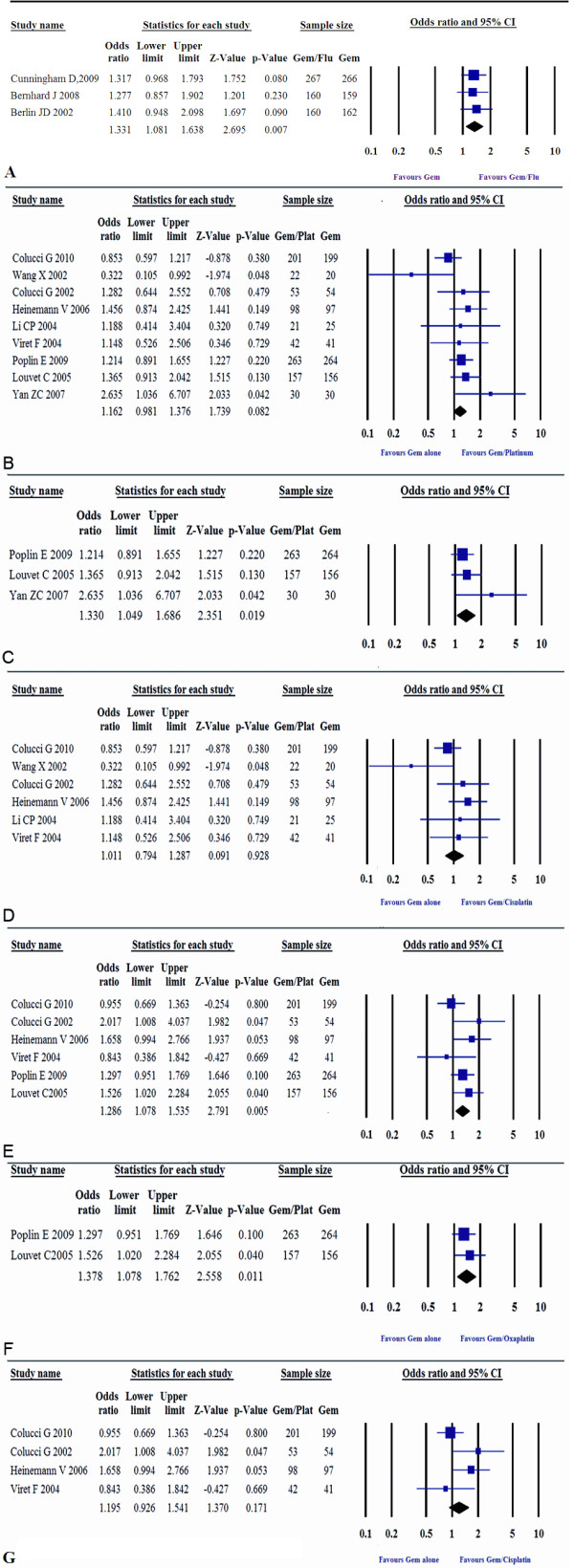
**Comparison of gemcitabine plus fluoropyrimidine or platinum with gemcitabine alone on OS and PFS**. A, gemcitabine/fluoropyrimidine versus gemcitabine alone on OS; B, gemcitabine/platinum versus gemcitabine alone on OS; C, gemcitabine/oxaliplatin versus gemcitabine alone on OS; D, gemcitabine/cisplatin versus gemcitabine alone on OS; E, gemcitabine/platinum versus gemcitabine alone on PFS; F, gemcitabine/oxaliplatin versus gemcitabine alone on PFS; G, gemcitabine/cisplatin versus gemcitabine alone on PFS.

Our analysis showed a significant improvement in OS (ORs, 1.33; 95% CI, 1.08 to 1.64; p = 0.007) (Figure 3A), PFS (ORs, 1.53; 95% CI, 1.24 to 1.88; p = 0.000) and ORR (ORs, 1.47; 95% CI, 1.04 to 2.07; p = 0.03) when gemcitabine was combined with fluoropyrimidine. The ORs for 1-year survival in the gemcitabine plus fluoropyrimidine group as compared with the group that received gemcitabine alone was 1.08 (95% CI, 0.82 to 1.43; p = 0.58).

### Trials comparing gemcitabine alone with gemcitabine plus platinum

The combination of gemcitabine with platinum was evaluated in eleven trials involving 2,379 patients. Three trials used oxaliplatin (Louvet 2005, Poplin 2009, Yan 2007), and eight trials (Colucci 2010, Colucci 2002, Wang 2002, Heinemann 2006, Palmer 2007, Li 2004, Kulke 2009, Viret 2004) used cisplatin combined with gemcitabine. In these trials, the gemcitabine/platinum combinations prolonged OS in nine trials, whereas no survival benefit was seen in two trials (Colucci 2010, Wang X 2002).

**Figure 4:**
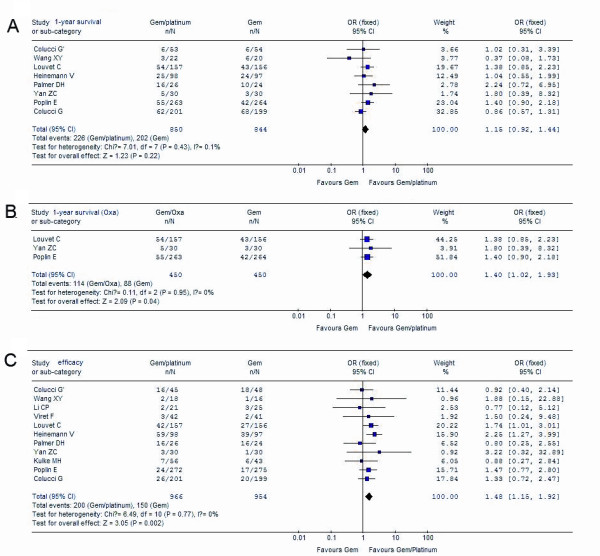
**Comparison of gemcitabine plus platinum combination with gemcitabine alone**. A, gemcitabine/platinum versus gemcitabine alone on 1-year survival; B, gemcitabine/oxaliplatin versus gemcitabine alone on 1-year survival; C, gemcitabine/platinum versus gemcitabine alone on ORR.

Meta-analysis showed that the combination of gemcitabine with platinum resulted in a significant improvement in PFS (ORs, 1.29; 95% CI, 1.08 to 1.54; p = 0.005) (Figure 3E) as compared with gemcitabine in monotherapy, though no statistical significant difference in OS was observed (ORs, 1.16; 95% CI, 0.98 to 1.38; p = 0.08) (Figure 3B). When ORR was compared, the platinum combination arm showed significantly higher disease control, which was reflected by a pooled ORs of 1.48 (95% CI, 1.15 to 1.92; p = 0.002) in favor of the platinum combination (Figure 4C.).

Subgroup analysis comparing the gemcitabine/oxaliplatin group with the gemcitabine alone group gave an ORs of 1.33 (95% CI, 1.05 to 1.69) for OS and ORs of 1.38 (95% CI, 1.08 to 1.76) for PFS, which was statistically significant (p = 0.019, p = 0.011, seperately) in favor of gemcitabine/oxaliplatin combination (Figure 3C, F). However, the comparison of gemcitabine/cispiatin with gemcitabine alone showed that there was no survival benefit (OS: ORs, 1.01, p = 0.93; PFS: ORs, 1.19, p = 0.17) (Figure 3D, G). There was also a trend toward to increased ORR in the gemcitabine/cisplatin combination versus gemcitabine alone, with a pooled ORs of 1.38 (95% CI, 1.00 to 1.91), but the difference was not significant (p = 0.05). With regards to one-year survival, we did not find a difference between the gemcitabine/platinum group versus gemcitabine alone (OR, 1.15; 95% CI, 0.92 to 1.44; p = 0.22) (Figure 4A), but there was a significant improvement in the gemcitabine/oxaliplatin group (OR, 1.40; 95% CI, 1.02 to 1.93; p = 0.04) in the subgroup analysis (Figure 4B).

One trial (Palmer 2007) compared gemcitabine plus cisplatin with gemcitabine in the neoadjuvant setting. The study showed that the percentage of patients who underwent resection was 38% in gemcitabine arm versus 70% in the combination arm, with no increase in surgical complications. The 12-month survival percentages for the gemcitabine and combination groups were 42% and 62%, respectively. Combination therapy with gemcitabine and cisplatin was associated with a higher resection rate and an encouraging survival rate, suggesting that further study is warranted.

### Trials comparing gemcitabine alone with gemcitabine plus camptothecin

**Figure 5:**
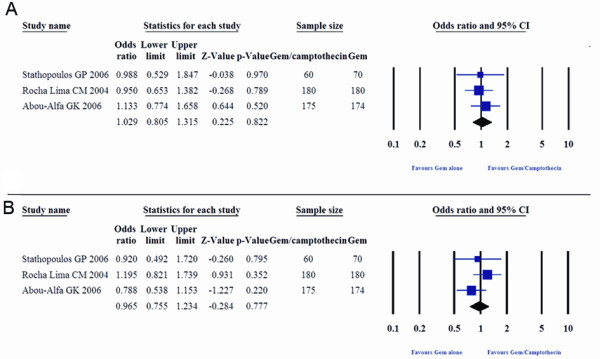
**OS and PFS of gemcitabine/camptothecin combination as compared with gemcitabine in monotherapy**. A, OS; B, PFS.

Four randomized trials (n = 839) compared the combination of gemcitabine and topoisomerase I inhibitors (irinotecan or exatecan) with gemcitabine monotherapy. They included three studies (Kulke 2009, Stathopoulos 2006, Rocha Lima 2004) in which gemcitabine was combined with CPT-11 (irinotecan) and one study (Abou-Alfa 2006) in which gemcitabine was combined with exatecan. The analysis revealed a significant improvement in ORR for gemcitabine plus camptothecin therapy (ORs 2.03; 95% CI, 1.28 to 3.23; p = 0.003; heterogeneity, p = 0.14). However, the combination did not significantly improve OS or PFS. The pooled ORs for OS and PFS were 1.03 (95% CI, 0.81 to 1.32; p = 0.82) and 0.97 (95% CI, 0.76 to 1.23; p = 0.78), respectively (Figure 5).

### Trials comparing gemcitabine monotherapy with gemcitabine plus other agents

Various other cytotoxic agents have been tested in combination with gemcitabine in LA/MPC patients, including pemetrexed (Alimta) and docetaxel. The analysis included two trials (n = 665), which indicated that the OS in the combination group was even lower than gemcitabine monotherapy (ORs, -0.10; 95% CI, -0.16 to -0.04; p = 0.002), although the ORR analysis showed therapeutic benefit of the combination (ORs, 1.91; 95% CI, 1.16 to 3.16; p = 0.01) (Figure 5B).

Oettle's trial, a randomized phase III study with 565 patients comparing the combination of gemcitabine and pemetrexed to gemcitabine alone, showed that OS was not improved in the combination arm (6.2 months) compared with the gemcitabine alone group (6.3 months) (p = 0.8477), although tumor response rate (14.8% versus 7.1%; p = 0.004) was significantly better in the combination arm.

### Trials comparing gemcitabine monotherapy with gemcitabine plus targeted therapy

The role of new, targeted drugs in the treatment of advanced pancreatic adenocarcinoma has been actively explored in the past few years. There are preliminary results and ongoing studies with EGFR inhibitors (erlotinib, cetuximab), farnesyltransferase inhibitors (tipifarnib), leukotriene B4 receptor antagonists (LY293111), antiangiogenic agents (axitinib, cilengitide), matrix metalloproteinase inhibitors (marimastat), vascular endothelial growth factor A inhibitors (bevacizumab), and histone deacetylase inhibitors (CI-994). However, most of these trials showed negative results.

In the present analysis, nine trials including 3, 342 patients evaluated gemcitabine combined with targeted therapy (Table 3). Although the results of the most recent trials (Philip 2010, Kindler 2010) are now available, which evaluated gemcitabine combined with C-225 or bevacizumab, so far Moore's trial is still the only study to demonstrate a significant improvement in survival in LA/MPC as a result of adding a targeted agent to gemcitabine. Therefore, the addition of other targeted agents is not recommended for the treatment of LA/MPC in the current clinical setting outside of a clinical trials.

**Table 3:** Median OS and DFS in trials comparing gemcitabine combined with targeted therapy with gemcitabine alone.

Trial	Regimen (per arm)	No. of pts	Median OS (mons)	HR (95% CI)	p value	Median PFS/TTP (mons)	HR (95%CI)	p value
Moore MJ 2007	Gem/erlotinib	285	6.24	0.82	0.038*	3.75	0.77	0 .004*
	Gem	284	5.91	(0.69-0.99)		3.55	(0.64-0.92)	
Philip A 2010	Gem/C-225	372	6.3	1.06	0 .23	3.4	1.07	0.18
	Gem	371	5.9	(0.91-1.23)		3.0	(0.93-1.24)	
Van Cutsem E 2004	Gem/tipifarnib	341	6.4	1.03	0 .75	3.7	1.03	0 .72
	Gem	347	6.1	(0.86-1.23)		3.6	(0.87-1.22)	
Saif W 2009	Gem/LY293111	67	7.1	UA	> 0.05	3.7	UA	> 0.05
	Gem	66	8.3			3.4		
Spano JP 2008	Gem/axitinib	69	6.9	0·71	UA	4.2		UA
	Gem	34	5.6	(0.44-1.13)		3.7	(0.43-1.45)	
Bramhall SR 2002	Gem/marimastat	120	5.5	0.99	0.95	3.1	0.68	0.68
	Gem	119	5.5	(0.76-1.30)		3.2	(0.73-1.23)	
Kindler HL 2010	Gem/bevacizumab	302	5.8	1.004	0.95	3.8	UA	0.075
	Bev	300	5.9	(0.88-1.24)		2.9		
Richards DA 2006	Gem/CI-994	85	6.5	0.980	0.904	3.1	0.837	0.304
	Gem	88	7.1	(0.701-1.370)		3.4	(0.596-1.175)	
Friess H 2006	Gem/cilengitide	46	6.8	UA	> 0.05	3.7	UA	> 0.05
	Gem	43	7.8			3.8		

### Trials discussing gemcitabine doublets plus a third targeted reagent

Two trials (Cascino 2008, Vervenne 2008) including 691 patients evaluated a gemcitabine doublet with or without a third targeted reagent. In Cascino's multicenter randomized phase II trial, the addition of cetuximab to the gemcitabine/cisplatin combination did not increase PFS (hazard ratio 0.96, 95% CI, 0.60-1.52, p = 0.847) or OS (hazard ratio 0.91, 95% CI, 0.54-1.55, p = 0.739). In 2008, Vervenne compared the efficacy and safety of adding bevacizumab to erlotinib and gemcitabine in patients with metastatic pancreatic cancer. The results showed that addition of bevacizumab to erlotinib and gemcitabine did not significantly prolong OS, but there was a significant improvement in PFS (p = 0.0002). This combination requires further investigation in larger-scale clinical trials to assess efficacy and cost effectiveness.

Pooled analysis revealed slightly better disease control by adding a third reagent to the gemcitabine doublet, with an ORs of 1.62 (95% CI, 1.00 to 2.62), but this was not statistically significant (p = 0.05). Furthermore, the OS observed in the triplet group was disappointing (ORs, -0.79; 95% CI, -0.90 to -0.60; p < 0.00001).

## Discussion

Pancreatic adenocarcinoma is among the most challenging of solid malignancies to treat on account of its propensity for late presentation with inoperable disease, aggressive tumor biology and resistance to chemotherapy [43,44]. Gemcitabine monotherapy has become a cornerstone of therapy for patients with LA/MPC since Burris et al reported their phase III trial results. Although it has shown clinical benefit, gemcitabine monotherapy has been associated with limited antitumor activity, with an ORR of 5% and median OS of 5.7 months [5]. In the past decade, many randomized controlled trials evaluated gemcitabine combined with various cytotoxic or targeted agents to try to improve outcomes for patients with LA/MPC. Some of these studies have reported improved median OS and one-year survival rates. However, the question of whether gemcitabine-based combinations are better than gemcitabine monotherapy is still unclear.

To compare the efficacy and tolerability of gemcitabine-based combinations in the treatment of LA/MPC with sufficient statistical power, we performed this meta-analysis to overcome the statistical limitations (for instance, low case load) of the individual trials and investigate treatment efficacy, safety profile and survival benefit of various therapeutic combinations.

The systematic review yielded five major findings. First, the analysis showed that gemcitabine-based combination was associated with significantly greater OS (ORs, 1.15; p = 0.011), PFS (ORs, 1.27; p < 0.001), and ORR (ORs, 1.58; p < 0.001) than gemcitabine monotherapy. The study reported by Heinemann revealed similar results [45]; their study considered 15 trials including 4465 patients for the analysis of OS. The analysis revealed a significant survival benefit for gemcitabine+X (X = cytotoxic agent) with a pooled ORs of 0.91 (p = 0.004) and indicated that patients with a good PS had a marked survival benefit when receiving combination chemotherapy (ORs, 0.76; p < 0.0001).

A similar advantage for gemcitabine combined with fluoropyrimidine was observed in terms of the OS (ORs, 1.33; p = 0.007), PFS (ORs, 1.53; p < 0.001), and ORR (ORs 1.47, p = 0.03) as compared to gemcitabine alone. Although the occurrence of hematological toxicities, including neutropenia (ORs, 1.60; p = 0.002) and thrombocytopenia (ORs, 1.52; p = 0.04), was higher in the combination group, the incidence of anemia (ORs, 0.97; p = 0.90) and non-hematological toxicities such as nausea/vomiting (ORs, 1.10; p = 0.60) were similar in both groups.

It remained to be determined whether the combination of gemcitabine with 5-FU or that with capecitabine was better. Bolus 5-FU and high-dose leucovorin have not shown meaningful therapeutic benefits in phase II studies [46]. However, researchers have speculated that continuous-infusion of 5-FU could improve its therapeutic efficacy. Capecitabine (N4-pentyloxycarbonyl-5'-deoxy-5-fluorocytidine) (Xeloda; F. Hoffmann-La Roche, Basel, Switzerland), an oral tumor-selective fluoropyrimidine, has been reported to be as efficacious as continuous-infusion 5-FU. Capecitabine appears to be a reasonable substitute for infused 5-FU/LV in combination regimens or as monotherapy, with the added advantage of reducing the inconvenience of long infusion times [47]. Cartwright [48] reported that capecitabine alone has an ORR of 7.3% and a disease control rate of 24% in previously untreated patients with LA/MPC. In Cunningham's report, gemcitabine/capecitabine significantly improved ORR (19.1% v 12.4%; p = 0.034) and PFS (hazard ratio, 0.78; 95% CI, 0.66 to 0.93; p = 0.004) and was associated with a trend toward improved OS (hazard ratio, 0.86; 95% CI, 0.72 to 1.02; p = 0.08) compared with gemcitabine alone. On the basis of these results, he recommended that gemcitabine/capecitabine should be considered one of the standard first-line options in LA/MPC.

In 1993, Wils [49] was the first to report that single-agent cisplatin has therapeutic activity in LA/MPC with an ORR of 21%. Soon afterwards, several phase II studies discussed the gemcitabine plus cisplatin combination in a variety of schedules. Adding cisplatin to gemcitabine appeared to be very active, with ORR ranging from 9% to 31% and median OS from 5.6 to 9.6 months in these phase II trials [50-52]. Furthermore, in the neoadjuvant setting, Palmer (2007) showed that combination therapy with gemcitabine and cisplatin was associated with a high resection rate and an encouraging survival rate.

However, the pooled analysis showed that the PFS and ORR achieved with the gemcitabine and platinum combination were significantly greater than those achieved with gemcitabine monotherapy; however, no statistically significant difference between the 2 treatment approaches were observed in the case of OS. This result was consistent with that obtained in a study conducted by Bria [4]. In Bria's meta-analysis, platinum combinations led to the greater absolute benefits in terms of PFS and ORR as compared with single-agent gemcitabine (10% and 6.5%, respectively), but did not result in an OS benefit. However, Heinemann [45] reported contrary results, with a ORs of 0.85 (p = 0.010) for platinum-gemcitabine combinations compared to gemcitabine alone. Heinemann's study included 15 trials with 4465 patients, whereas Bria's study included 20 trials with 6296 patients; our study included 35 trials with 9979 patients. The greater number of included trials and case load in our study may have contributed to the more favorable results obtained in our study.

Further, our subgroup analysis showed that the OS (p = 0.019), PFS (p = 0.011), and one-year survival (p = 0.04) in the gemcitabine-oxaliplatin group were significantly better than those in the gemcitabine monotherapy group. On the contrary, the comparison of gemcitabine-cisplatin with gemcitabine alone showed that there was no survival benefit (OS: p = 0.93; PFS: p = 0.17) with the former. Hence, we concluded that the combination of gemcitabine and oxaliplatin is superior to gemcitabine plus cisplatin and may be recommended as one of the standard first-line therapies for LA/MPC.

The third key finding was that the combination of gemcitabine plus other cytotoxic agents showed disappointing results. According to the literature, the combination of gemcitabine and irinotecan resulted in an objective response of 25% with a median OS ranging from 5.7 to 7 months (Rocha-Lima 2002, Stathopoulos 2004). Although our analysis found an enhanced ORR for gemcitabine plus camptothecin therapy (ORs, 2.03; p = 0.003), we did not find a significant difference in OS (ORs, 1.03; p = 0.82) or PFS (ORs, 0.97; p = 0.78) in the comparison. Other single agents including docetaxel and pemetrexed have also been tested in advanced pancreatic cancer. However, the analysis of two trials (n = 665) showed negative results. The OS in the combination group was even lower than that of patients receiving monotherapy (ORs, -0.10; p = 0.002), although the ORR analysis showed therapeutic benefit for this combination group (ORs, 1.91; p = 0.01).

Fourth, the identification of novel targets is still elusive for the treatment of LA/MPC. Since 2002, there has been a series of disappointing results. The only exception is erlotinib, which is the first and only targeted agent to demonstrate significantly improved survival in advanced pancreatic cancer when added to gemcitabine. Further research should be focused on new combinations or multi-target combined therapy, incorporating new, targeted therapies and identifying potential predictive factors of response.

The fifth finding concerned combining a gemcitabine doublet with or without a third targeted reagent. Our analysis revealed slightly better disease control by adding a third reagent to a gemcitabine doublet, with an ORs of 1.62 (95% CI, 1.00 to 2.62), but this difference was not statistically significant (p = 0.05). The OS in the triplet group was also disappointing (ORs, -0.79; p < 0.00001). Vervenne's study showed that addition of bevacizumab to erlotinib and gemcitabine did not significantly prolong OS, but there was a significant improvement in PFS (p = 0.0002). This suggested that multi-target therapy may be a future direction for the treatment of advanced pancreatic cancer. This combination should be further evaluated in larger clinical trials to assess its efficacy and cost effectiveness.

The present meta-analysis was not based on individual patient data and was not subjected to an open external-evaluation procedure. Therefore, the analysis is limited in that the use of published data may have led to an overestimation of the treatment effects. Although the risk of publication bias exists in any meta-analysis, we believe that this did not greatly affect our results because many positive and negative trials were included in the study.

Moreover, some trials investigated gemcitabine-free combinations such as irinotecan/docetaxel or FOLFIRINOX for the treatment of LA/MPC. Among them, FOLFIRINOX (5-FU/leucovorin, irinotecan, and oxaliplatin) is an interesting and promising combination. At the 2007 ASCO annual meeting, Ychou reported that the use of FOLFIRINOX as the first-line treatment for advanced pancreatic cancer afforded a response rate of greater than 30% with manageable toxicity in ECOG 0-1 patients [53]. In another study, Breysacher discussed the role of FOLFIRINOX as second-line therapy for metastatic pancreatic cancer [54]. No response was seen in 13 patients, and the one-year survival rate was 62%. However, a large-scale randomized clinical trial is required to evaluate the efficacy of FOLFIRINOX.

In the end, the goals of treatment for advanced pancreatic cancer should be to control tumor progression, alleviate disease-related symptoms and improve and maintain patients' quality of life (QOL). Reni [55] reported on the effects of a gemcitabine combination versus monotherapy on patient QOL. The study showed that the largest differences between arms favored the gemcitabine combination group. Clinically relevant improvement in QOL from baseline was observed more often after combination therapy than after gemcitabine, suggesting that the combination regimen did not impair QOL.

## Conclusion

In general, the benefits of adding capecitabine or oxaliplatin to gemcitabine chemotherapy in LA/MPC are clear, with prolonged survival, improvement in disease control and improvement or stabilization of QOL as compared with gemcitabine monotherapy.

## Competing interests

The authors declare that they have no competing interests.

## Authors' contributions

HJ, TL and XYC performed computerized search of trials, contacted experts and participated in the trials selection. MY and ZG participated in the trials selection and performed the statistical analysis. WHX conceived of the study and drafted the manuscript. All authors read and approved the final manuscript.
